# Vision-Based Sensor for Three-Dimensional Vibrational Motion Detection in Biological Cell Injection

**DOI:** 10.3390/s19235074

**Published:** 2019-11-20

**Authors:** Ferhat Sadak, Mozafar Saadat, Amir M. Hajiyavand

**Affiliations:** Department of Mechanical Engineering, School of Engineering, University of Birmingham, Birmingham B15 2TT, UKm.saadat@bham.ac.uk (M.S.)

**Keywords:** intracytoplasmic sperm injection (ICSI), 3-D vibration, computer vision, image processing, egg deformation, vision sensor

## Abstract

Intracytoplasmic sperm injection (ICSI) is an infertility treatment where a single sperm is immobilised and injected into the egg using a glass injection pipette. Minimising vibration in three orthogonal axes is essential to have precise injector motion and full control during the egg injection procedure. Vibration displacement sensing using physical sensors in ICSI operation is challenging since the sensor interfacing is not practically feasible. This study proposes a non-invasive technique to measure the three-dimensional vibrational motion of the injection pipette by a single microscope camera during egg injection. The contrast-limited adaptive histogram equalization (CHALE) method and blob analyses technique were employed to measure the vibration displacement in axial and lateral axes, while the actual dimension of the focal axis was directly measured using the Brenner gradient algorithm as a focus measurement algorithm. The proposed algorithm operates between the magnifications range of 4× to 40× with a resolution of half a pixel. Experiments using the proposed vision-based algorithm were conducted to measure and verify the vibration displacement in axial and lateral axes at various magnifications. The results were compared against manual procedures and the differences in measurements were up to 2% among all magnifications. Additionally, the effect of injection speed on lateral vibration displacement was measured experimentally and was used to determine the values for egg deformation, force fluctuation, and penetration force. It was shown that increases in injection speed significantly increases the lateral vibration displacement of the injection pipette by as much as 54%. It has been demonstrated successfully that visual sensing has played a key role in identifying the limitation of the egg injection speed created by lateral vibration displacement of the injection pipette tip.

## 1. Introduction

Physical sensors are commonly used to measure the displacement of the objects in the micro-world in various applications such as micro-manipulation and micro-gripping [1,2]. However, these types of sensors have limited measurement distance and are sensitive to object spot size and the object’s material. Existing micromanipulators for biological egg injection are not equipped with any displacement sensors as it is not possible to interface due to the inappropriate assembly location which causes a high amount of noises interacting with the real data. Considering the limitation of the sensor accommodation in intracytoplasmic sperm injection (ICSI), interfacing three sensors for each access would not practically feasible. On the other hand, since the ICSI operation is conducted when the egg and sperm are in an aqueous medium, this affects the accurate measurement of the displacements for both contact and-non-contact physical sensors. Additionally, sensor interfacing greatly increases the cost of micromanipulation setup. Vision-based displacement sensing is more suitable to use in an aqueous medium [3]. The outstanding advantages of the vision-based displacement sensor are its low cost, ease to implement, high reliability and accuracy [4] and measurement of displacement in three orthogonal axes at once by a single camera. 

Automated microinjection has demonstrated rapid improvement in various fields such as ICSI which offers a precise motion for the injection pipette during egg injection [5]. Injection speed and trajectory of the injection pipette are two main injection parameters that contribute toward a successful egg injection [6]. The displacement of the injection pipette is affected significantly by cell injection speed and its trajectory [7,8]. Besides, the magnitude of the vibration at the tip of the injection pipette increases since the injection procedure requires acceleration and deceleration of the injection pipette [9]. Produced vibration at the tip of the injection pipette is out of control and negatively affects the accuracy of the egg injection operation. The injection pipette is driven by a motorized stage during egg injection and the resolution of the motorized stage is not effectively utilized if the undesired displacement changes are not measured correctly. Regardless of the amount of vibration induced at the tip of the injection pipette in any direction during egg injection, uncontrolled vibration is an undesired dynamic factor which may affect the control strategies developed, particularly in the ICSI operation, such as position and force control [10]. On the other hand, induced undesirable vibration at the tip may cause damage to the membrane of the oocyte and lead to degeneration. If this damage cannot be healed effectively, abnormal growth occurs and this causes the failure of the operation [11,12]. Overall, vibration is an undesired dynamic factor preventing the injection operation to be conducted in a stable environment. Therefore, minimizing the vibration at the tip of the injection pipette is essential in the ICSI operation. 

Analysis of vibration in the ICSI procedure is very challenging due to the complexity of the ICSI machine and the impractical use of displacement sensors. Analytical and numerical models have been proposed in the literature to analyse the vibration at the drawn section of the injection pipette [13]. There are various sources of internal and environmental parameters which induce the vibration on the injection pipette. Hence, it is not safe to rely only on numerical and analytical analysis as it does not cover all existing dynamic parameters which affect the displacement of the injection pipette tip. In another study, three photonic displacement sensors were implemented into the cell manipulation system to measure the vibration at the tip of the injection pipette holder for each vibrational direction as reported in [14]. However, due to the tiny diameter of the injection pipette tip, the injection pipette could not be present in their experiment; instead, it has been detected manually in 2 dimensions by using a high-speed camera.

A vision-based sensor for vibrational detection of the injection pipette tip in three dimensions requires an accurate tip recognition of the injection pipette. Image-based detection of the injection pipette tip position in the X, Y axes and focus estimation in the Z-axis is one of the steps to automate the ICSI process [15]. As the injection pipette must be located under the microscope prior to the cell injection procedure, Wu et al. proposed a vision-aided injection pipette tip detection algorithm [16]. In their study, Canny edge detection and Hough transform were utilized for the detection of the injection pipette tip. However, the disadvantage of using the Hough transform is that it can only be applied if the object has a regular shape, such as lines or circular shapes [17,18]. The template-matching technique has also been proposed for micro-object tip tracking purposes in micro-robotics applications [5,19]. However, locating micro-objects using the template matching technique is not an efficient way since this method requires clear morphology [15]. Then again, using a template matching method is also computationally expensive [20]. Li et al. developed a technique to detect the micro-motion of the 3 degrees of freedom (DOF) precision positioning stage and then a nanometre level accuracy was achieved by the proposed micro-vision imaging system to extract the in-plane displacements data [21,22]. Zhang et al. developed a robust rotation-invariant displacement method for the micro-nano positioning system [23,24]. Theoretically, 0.001-pixel accuracy was achieved in their study. However, none of this type of vision-based precision positioning stage micro-motion detection helps to measure the injection pipette displacements in three orthogonal axes in ICSI operation and, in particular, it does not provide information in focal axis changes at the tip of the micropipette which makes accurate vibration displacement measurement in three-dimensional space a necessity in the field of ICSI. 

This paper proposes a method to sense the vibration displacement visually during egg injection in three orthogonal axes. Afterward, the information obtained by the developed image-processing algorithm is utilized to find the displacement of the injection pipette tip. Subsequently, the effect of egg injection speed on vibration creation during injection was presented experimentally. The critical role of lateral vibration displacement and its measurement was presented. To the best of our knowledge, this is the first vision-based sensor proposed in the literature to aid the reliability of ICSI operation and track the displacement of the injection pipette tip visually in three orthogonal axes. 

## 2. Materials and Methods

This section is introducing the developed measurement technique for the three-dimensional vibration displacement of the injection pipette during egg injection. The developed measurement technique is divided into 2 main sections. The first part of the image-processing algorithm measures the tip displacement in lateral and axial directions while the other part of the algorithm calculates the displacement in the focal axis. A vibration term was used precisely to explain the mechanical oscillation of the tip of the micropipette. In the following section, primarily, the system configuration will be explained.

### 2.1. System Configuration and Calibration

**Figure 1:**
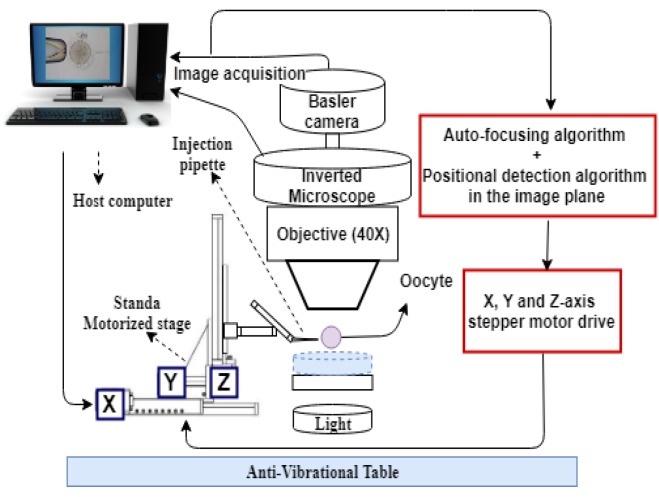
Schematic diagram of the cell-injection system setup.

Figure 1 illustrates the schematic diagram of the three-dimensional egg injection setup. This setup is composed of an inverted microscope (Best-scope 2090), and a microscopy camera (BASLER Camera acA1300-200Um - Python1300), which can take up to 200 frames per second. The injection unit is firmly assembled on a 3-DOF motorized stage (Standa 8MT167) with an accuracy of 1 µm to move the injection pipette towards the desired positions using the BASLER camera as a vision sensor. The positional control of the motorized stage and image processing algorithms are hosted by a computer. Captured images are processed by an Intel® Core™ i5-6500 CPU @3.2 GHz (4 CPUs) host computer. The entire trajectory motion of the injection pipette during injection is captured and transferred to the host computer as a series of frames. Each frame is then analysed by the in-house developed image-processing algorithm in three orthogonal axes. 

**Figure 2:**
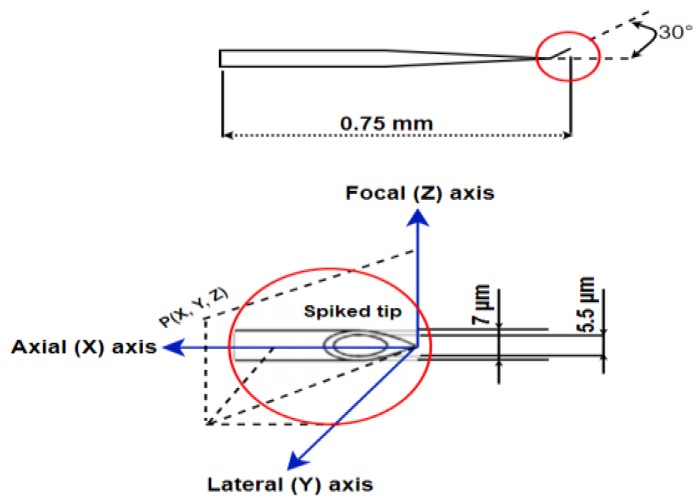
The dimensions of the injection pipette and its schematic 3D view.

The dimensional detail of the injection pipette used for the cell-penetration operation as well as its schematic 3D view is given in Figure 2. 

The technique developed for the positional detection of the injection pipette in axial and lateral axes requires system calibration before the injection procedure starts. In this study, the outer diameter of the injection pipette is utilized as a dimensional reference to find the pixel size by using ImageJ software. The pixel size obtained will be employed into the developed Matlab algorithm as an input to report the positional changes in the X and Y axes versus time in real-world dimensions

### 2.2. Detection of Positional Changes in Image Plane

In this section, the algorithm development procedure will be presented for extracting the 2-dimensional vibrational displacement information of the injection pipette tip. The steps involved in the development of the image-processing algorithm are pre-processing, filtering and thresholding, edge detection, and tip position measurement of the injection pipette. The detail of these steps will be presented and discussed in the following part of the study. 

• **Pre-Processing**

**Figure 3:**
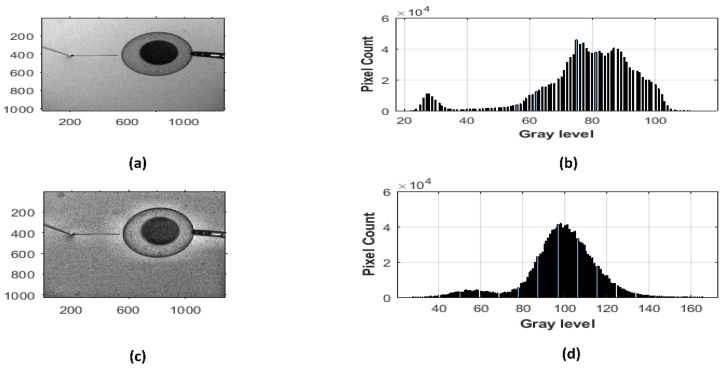
Enhancement of gray-scale intracytoplasmic sperm injection (ICSI) image by the contrast-limited adaptive histogram equalization (CHALE) method and its histograms (**a**) Original ICSI image; (**b**) histogram of the original ICSI image; (**c**) contrast-limited adaptive histogram equalization (CLAHE) image; (**d**) histogram of CHALE image.

The acquired images captured by the microscope camera require pre-processing for its further analysis. To increase the efficiency of the developed algorithm, the size of all the images acquired during egg injection was reduced to 50%. The captured image is in the eight-bit grayscale type where intensity level varies from 0 to 255. RGB, which stands for Red, Green and Blue, images are converted to grayscale within the developed algorithm. A grayscale image is then used for the positional detection of the ICSI pipette. Contrast-limited adaptive histogram equalization (CLAHE) can only be applied to the grayscale image. CHALE is used to improve the contrast in images while avoiding amplifying the existing noise in the image. This is a particularly significant feature to highlight the ICSI pipette for its further analysis as the developed measurement technique relies on the detection of the ICSI pipette accurately. In this study, the CLAHE method is implemented as pre-processing. In CLAHE, the input image is divided into small rectangular areas, which is called tiles, and then the local histogram for each tile is adjusted by enhancing their contrast [25]. The histogram is a graphical way of showing the frequency of occurrence at different color intensities in the image. Each bar in the histogram represents the total number of pixels at a particular intensity level changing from 0 to 255; 0 indicates the black in the histogram while 255 indicates white. In generalized, a balanced gray level is desirable in the histogram, which makes peaks in the middle and tapers off towards the edges. The comparison of gray-level distribution for the image of the ICSI procedure and processed image by the CHALE method are shown in Figure 3.

The histogram of the original ICSI pipette image as demonstrated in Figure 3b illustrates that the image is overexposed. That means the input for image segmentation is not sufficient since the input image is not able to detect the edges in comparison to higher contrast input. On the other hand, the histogram of the image obtained by the CHALE method illustrates a much improved exposed image with evenly distributed gray-level bars and higher saturated tones. Hence, the CHALE method is implemented successfully into the developed image processing algorithm as pre-processing to not lose any data on the image and lead any failure of the injection pipette tip positional detection in axial and lateral axes.

• **Filtering and Thresholding**

Filtering is a significant step to enhance the performance of the developed algorithm. The level of noise increases during the image acquisition and transmission process. Hence, it is essential to reduce the negative effect of the noises [26,27]. A Gaussian filter is a linear filter that performs a weighted average of surrounding pixels chosen based on Gaussian function. Consequently, a Gaussian filter is utilized to reduce the noises on the ICSI image. The two-dimensional Gaussian filter used on ICSI image can be demonstrated in Equation (1).

(1) $$G\left(x,y\right)=\frac{1}{\sqrt{2\pi \sigma }}\exp\left(-\frac{x^{2}+y^{2}}{2\sigma^{2}}\right)$$

where σ indicate the standard deviation of the distribution for the Gaussian filter and x and y illustrate the distance from the origin in the horizontal and vertical axes, respectively. 

The grayscale image needs to be converted into the binary image as a part of the thresholding procedure. The binary image is presented only as 0 and 1 for the background pixel and the highest intensity in the image plane. Adaptive thresholding is a common solution when the variations in illumination is a consideration. In this method, the threshold value is computed for every single pixel. This algorithm calculates the thresholding value for a small region, and then it applies different thresholding values for the other region of the image. Therefore adaptive thresholding provides better robustness changes in illumination [28]. Hence, the adaptive image threshold is employed to obtain the binary image of the ICSI process. 

Binary images can be noisy and any scattered pixels are required to be removed from the image for further processing. The Matlab bwareafilt function is utilized to eliminate the small pixels around the injection when holding the pipette and a zebrafish egg. 

• **Edge Detection**

The result of the binary image is then used to obtain the contour of the entire ICSI process through Canny edge detection [29]. In this method, the input image is converted into a set of curves to extract the remarkable features on the image. These remarkable features are the contour of the objects in the plane that it is aimed to detect. This detection relies on sudden intensity changes in the image. Hence, initially obtained curves data are transformed into the lines at the end of the procedure. 

• **Measurement**

Blob detection is a method to detect the regions in the image by distinguishing properties such as mean intensity, area, diameter, perimeter, centroid, etc. in comparison to surrounding regions. Each blob is labelled in order to make a measurement on the ICSI process. In our case, in the ICSI process, the developed vision-based vibration-sensing technique detects two blobs, one is for injection pipette and one is for the combination of holding the pipette and the egg. The properties of these two blobs are extracted, which are mean intensity, area, perimeter, centroid, and diameter of each blob. Pixel intensity and the area of the blobs were sufficient to distinguish them from each other. The properties of the injection pipette, i.e. its area and pixel intensity, are defined within the developed Matlab algorithm to keep only the injection pipette on the image plane by isolating the rest of the objects which is the combination of holding pipette and the egg. These properties should be calibrated if the magnification of the microscope changes as it affects the value of the properties of the injection pipette. Afterward, the developed vision-based displacement sensor ignores any blobs which do match with the properties of the injection pipette and just keeps the injection pipette for further analysis. Subsequently, the far-right pixel of the binary image is detected and reported in the X and Y axis in pixels. The pixel size obtained from the calibration operation is employed to convert the pixel measurement into actual dimensions. 

**Figure 4:**
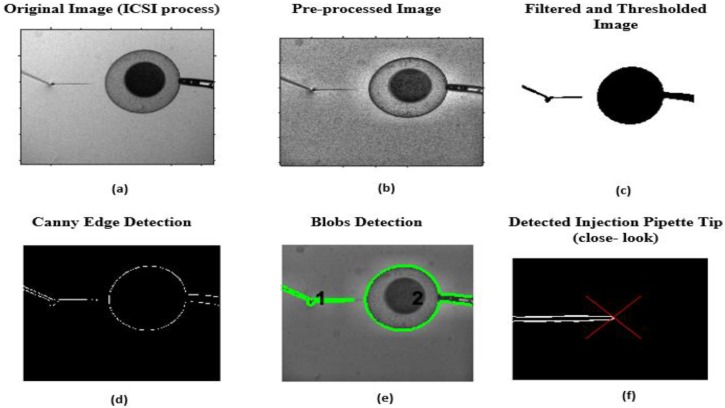
Steps in vision-based positional detection of the injection pipette: (**a**) original ICSI image captured by the camera; (**b**) image after implementation of the CLAHE method; (**c**) ICSI image after application of Gaussian filter and adaptive thresholding; (**d**) extraction of the contour information of the ICSI image using canny edge detection algorithm; (**e**) recognition of the egg and injection pipette and distinguishing them based on their blobs feature; (**f**) detection of the injection pipette tip indicated by red cross.

Figure 4 demonstrates the steps involved in the positional detection of the injection pipette tip in the image plane. 

### 2.3. The Detection of Focalization Position Changes

The technique for the focalization of the injection pipette tip has been conducted previously by our research group and the details can be found in [30]. In this study, the focalization procedure of the injection pipette tip is utilized to find the positional changes in Z-axis at the time of cell injection. In order to obtain the focal position of an object under a microscope, focus algorithms are widely used [31,32]. These algorithms provide information regarding the degree of focus for the number of images taken by varying the focus lens position. The Brenner gradient algorithm was selected as the focusing algorithm after the evaluation of the 12 different focus algorithms examined for this application. In the Brenner gradient algorithm, the square difference of each pixel between its two neighbours are calculated and then they are summed using Equation (2) [33].

(2) $$F_{brenner}=\underset{x,y}{\sum }i\left(x+1,\;y\right)-i\left(x-1,y\right){)}^{2}$$

Considering |i(x + 1), y) − i(x − 1), y)| > ɑ, where i (x, y) is the intensity at pixel (x, y), ɑ is the threshold of the intensity difference. 

To locate the injection pipette tip to the focal plane under a microscope, the movement of the injection pipette is scanned in Z-axis in a range between 0 to 34 µm with an increment of 2 µm. Hence, a set of 18 images was attained. The initial position presents the most blurred image under the focal plane, while the final destination position demonstrates the most blurred one above the focal plane. 

**Figure 5:**
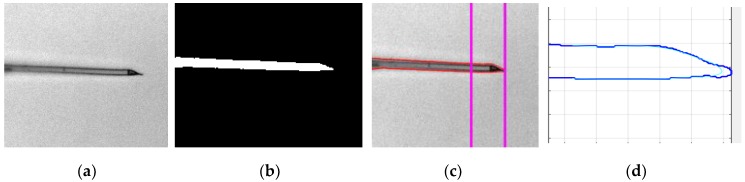
Region of interest extraction: (**a**) original injection pipette image; (**b**) final binary image; (**c**) image with a boundary at the ratio of 1:4 divided by lines; (**d**) extraction of the region of Interest.

Since the level of noise increases during the image acquisition process, it requires pre-processing to enhance the performance of the algorithm. The noise on the images is eliminated by using a Gaussian filter as a part of pre-processing. Then, the adaptive image threshold using first-order statistics is utilized and the binary image of the injection pipette was obtained. Obtaining a binary image of the injection pipette without any scattered points is essential since boundary extraction of the injection pipette if fully dependant on the obtained binary image. However, as it is visible in the obtained binary image of the injection pipette in Figure 5, at some fraction of the boundary of the injection pipette, irregular data points result. In order to prevent any irregular data points, particularly at the tip of the injection pipette, a kernel smoother was employed to set the irregular data points on the boundary of the injection pipette as a smooth line.

The extracted boundary is divided by two lines at the ratio of 1:4 starting from the tip of the injection pipette. The created mask in this area is considered as a region of interest for the image-processing algorithm. Finally, the Brenner gradient algorithm is implemented in the region of interest extracted. Figure 5 illustrates the marginal detection of the injection pipette image and its region of the interest extraction procedure. 

**Figure 6:**
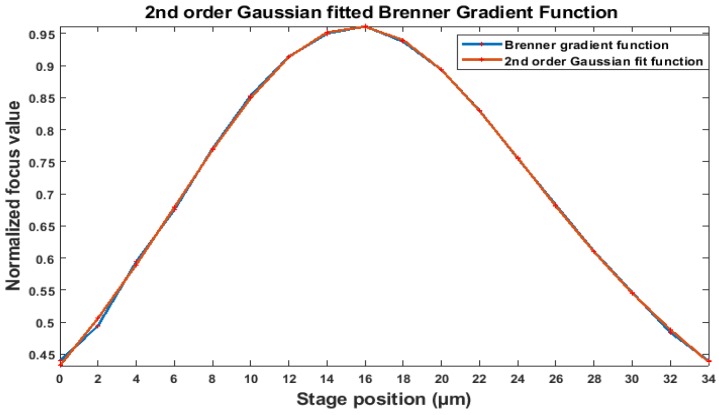
Representation of the focus curve obtained from the Brenner gradient method.

Figure 6 demonstrates the pattern of the normalized focus measurement values versus stage position. As it is shown, the injection pipette presents an unimodal curve that enables Brenner gradient algorithm to be utilized. Also, the maximum value of the normalized focus value corresponds to the sharpest image where the stage position is in the focal plane. Subsequently, the second-order Gaussian fit is implemented to the curves obtained from the Brenner gradient method for the injection pipette [34]. In order to search the global maximum of the curves precisely, a Fibonacci search algorithm is employed to the procedure into the defined focus range [35]. In this method, the range of search is defined for the algorithm and the focus range was narrowed successively until the focal plane is obtained. It has been proved that the Fibonacci search algorithm was found to be an optimal search algorithm under the assumption of the unimodal property of the focus curve [34]. Figure 6 clearly demonstrates that there is a peak where the tip of the injection pipette is entirely in focus.

In Figure 6, each stage position has got a corresponding normalized focus value. Initially, a set of images saved at the time of egg injection are analysed based on its normalized focus value. Each frame is labelled with a normalized focus value obtained from the Brenner gradient algorithm. The reliability of the Brenner gradient method at the same focal level at different XY plane was previously verified for this particular application in our previous publication [30]. Since the equation of the curve was obtained after the implementation of the Gaussian fit method, each focus value will have the corresponding stage position between 0 to 34 µm. As the above and below the focal plane is identical, by being independent to the direction selection, the right-hand side of the curve was taken into account for the implementation of the normalized focus values to be implemented into the focus function. Therefore, these normalized focus values are converted into the real dimension by the algorithm developed. Finally, each position of the frame in the Z-axis is subtracted from the focal point of the focus curve. The differences are reported as displacement in Z-axis versus time. This technique is capable of measuring the vibration displacement while the injection pipette is in the field of view under the microscope. Based on the assessment of the Brenner gradient algorithm in our previous work, this algorithm is reliable and efficient to report the same pipette position in Z-axis by being independent to the X and Y axes in the field of ICSI.

**Figure 7:**
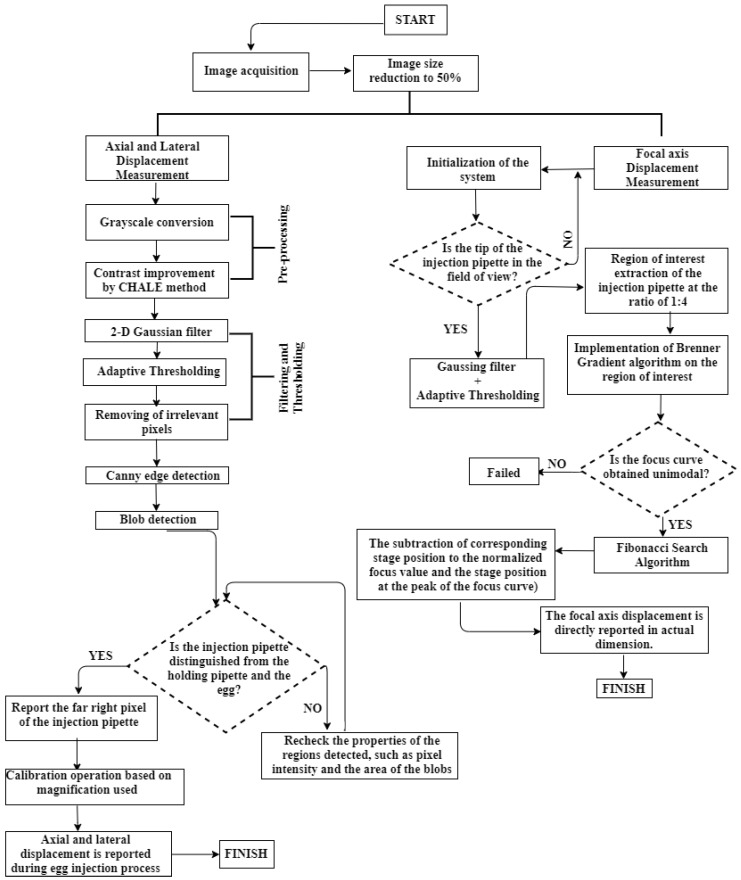
Block diagram of the vibration displacement sensing procedure by the developed vision algorithms in 3 dimensions.

Figure 7 demonstrates the block diagram of the developed algorithms to calculate the vibration displacement of the injection pipette tip in axial, lateral and focal axes. 

## 3. Results and Discussion

**Figure 8:**
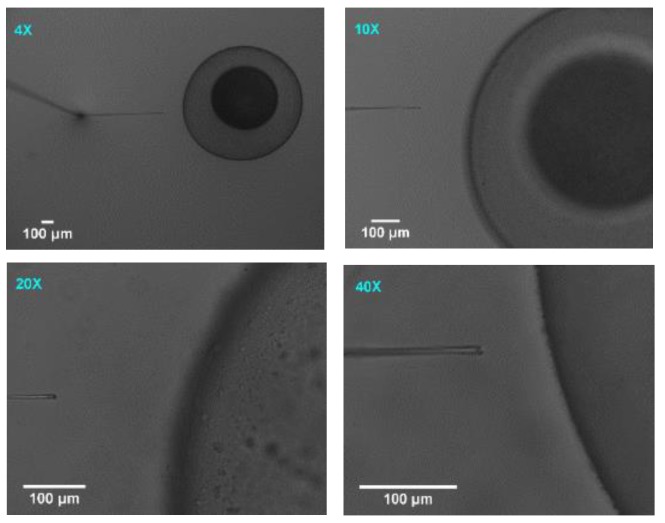
Egg-injection procedure at different magnifications.

In the previous sections, the method of a three-dimensional vision-based vibration displacement measurement method was demonstrated. Here, the algorithm is examined and the results are presented in the following section. For evaluating the functionality of the algorithm, the vibration was introduced randomly to the system to demonstrate the effectiveness of the algorithm developed. Subsequently, the motion of the injection pipette was analysed by the algorithm developed. The results obtained from the algorithm have then been validated by using ImageJ software. This software lets the user select the desired pixel value in the XY plane manually. Hence, the tip coordinate of the injection pipette in the XY plane was compared with the results obtained manually from ImageJ software. The total number of 100 images were captured sequentially during the egg injection. These image acquisitions procedures were conducted separately at four different magnifications, as shown in Figure 8. 

Before the experiment was conducted, the initial position of the injection pipette tip was recorded in each magnification. This value is subtracted from each reported coordinates from the vision algorithm during egg injection which took 0.5 seconds for all levels of magnification. Then, the magnitude of vibration displacement was measured and reported in axial and lateral axes by the developed vision algorithm.

**Figure 9:**
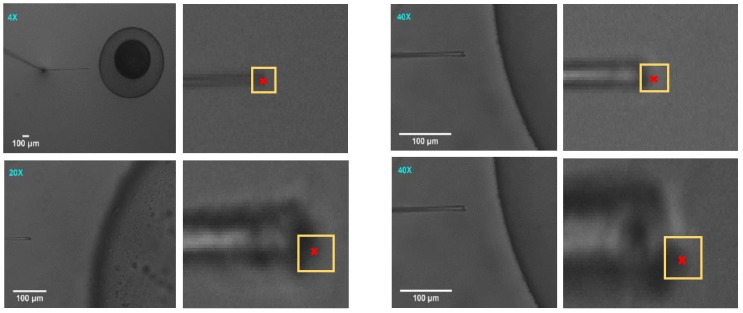
Pixel selection by ImageJ software.

To validate the results obtained from the developed algorithm, the ImageJ software is employed. This is a manual image processing program which is developed to analyse and process the images. For this reason, the images were focused on the pixel levels, and the final pixel which demonstrated the tip of the pipette was manually selected. Figure 9 illustrates the original and focused zoom used in ImageJ. Figure 9 illustrates that different magnifications provide different focused zoom. The yellow square shows the area of the interest for manual selection and the red cross on the images shows the point which was selected for the tip of the pipette. By increasing the magnification, the possibility of manual selection of the most appropriate pixel, which represents the tip of the pipette, increases. 

**Figure 10:**
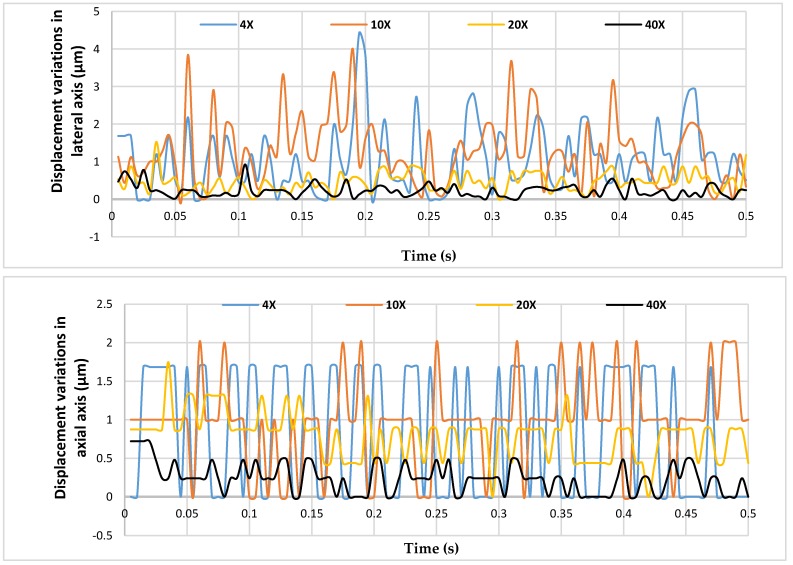
Displacement variations between developed vision sensor and Image J software in axial and lateral axes.

This method of comparison demonstrates the accuracy of the developed algorithm compared to manual selection. It is noted that there may be some human errors contributing to these results, however, integrating other types of physical sensors would not be practically possible. On the other hand, this comparison has higher validity due to matching the results based on an image which eliminates other environmental contributing factors. Figure 10 illustrates the differences in results obtained manually and by the developed algorithm both laterally and axially. 

The detail of the average axial and lateral vibration displacement variations at each magnification is shown in Table 1. The level of standard deviation is high as expected due to the vibration introduced.

As the comparison results demonstrate in Table 1, the variations between the developed algorithm and the ImageJ software were both decreased when the level of magnification increased. This is due to the changes in pixel sizes. Considering the total tip size which is 7 µm, the area of interest provides only 4 allowed pixels for manual selection in 4×, however, the same area of interest provides 42 allowed pixels in 40× which hugely decreases the potential errors of manual selection. This can be confirmed in Figure 10 as well which shows the variations decrease by 71% and 79% from 4× to 40× for axial and lateral vibration displacement, respectively. Overall, the developed vision algorithm demonstrated consistency and its reliability with the results obtained by ImageJ software.

**Figure 11:**
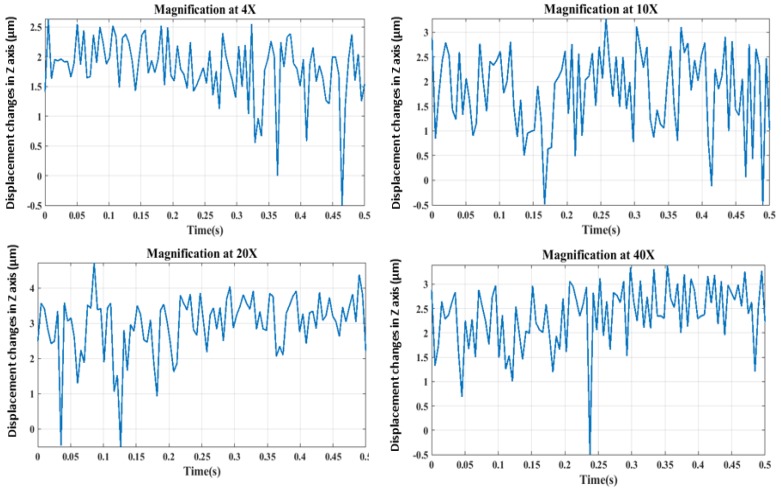
Injection tip position changes in Z-axis using the Brenner gradient method.

The displacement of the injection pipette tip in the Z-axis was obtained with the utilization of the Brenner gradient method. A number of 100 images were captured sequentially during injection at different magnifications. Later, their normalized focus value was introduced to the focus function obtained from the Brenner algorithm. The corresponding stage position for each focus value was obtained. As shown in Figure 6, the stage position at the peak of the curve is known. Therefore, the stage position at each frame was subtracted from the position where the tip position of the injection pipette has a peak in the curve. The displacement variations in Z-axis versus time were plotted at different magnifications in Figure 11.

As it is shown in Figure 11, the vibration-induced injection pipette position has changed in the Z-axis by its focus value at different magnifications. As previously stated, the right-hand side of the focus curve was used for the implementation of the normalized focus values which were implemented into the focus function. Hence, the resultant vibration displacement in the focal axis is only positive. The displacement of the injection pipette is directly obtained as shown in Figure 11 using the Brenner gradient algorithm as focus measurement. 

This study assists in the need for vibration-sensing process accurately in three orthogonal axes in injection procedures. Currently, the effect of vibration at the tip of the injection pipette in the ICSI procedure is not studied in the literature. This study will try to study the viability of the vibration analysis to realise the role of vibration displacement in three dimensions induced by any sort of internal and external dynamic parameters and whether that may contribute to the success rate of the ICSI operation. For this purpose, another set of experiments was conducted to demonstrate the key role of lateral vibration displacement in ICSI. 

**Figure 12:**
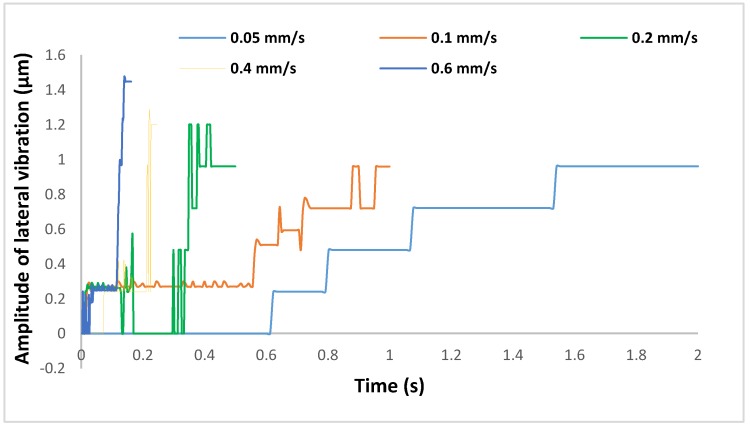
The effect of egg-injection speed on vibration creation.

As the algorithm had been validated, it was then used to evaluate the effect of egg-injection parameters such as speed on the vibration. In the previous publication, the authors demonstrated the variations in forces and deformation creation during egg injection [8]. In this research, the potential contribution of the demonstrated factors is evaluated in vibration creation which may be a cause of egg damage during the penetration. Injection speed was selected as the main contributing factor in potential damage to the cell. So, the various speeds were considered for the vibrational measurements. Then the results obtained were correlated to the previous results. The total travel distance was assigned to be 100 µm for each injection speed. Figure 12 illustrates the lateral vibrational displacement results at various speeds.

As illustrated in Figure 12, the injector starts with some vibrations, but it moves steadily. However, at the end of the motion procedure, the lateral vibration displacements dramatically increased. This moment is considered as the injection time. This is due to the acceleration and deceleration maneuver of the injector. On the other hand, the vibration displacements were increased by increasing the speed as expected in the hypothesis.

**Figure 13:**
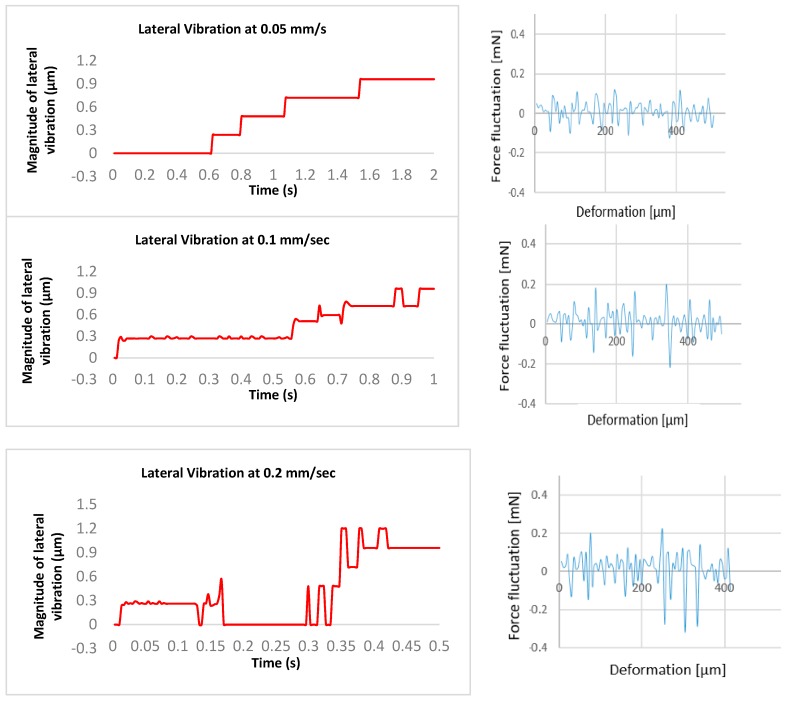

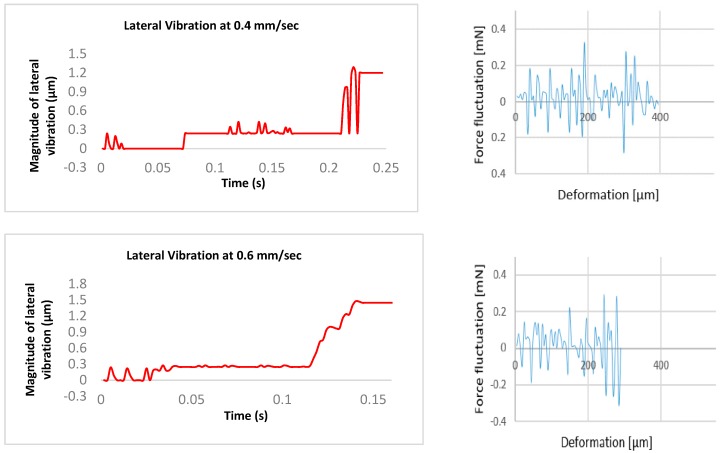
Lateral vibration displacement, force fluctuation, and egg deformation at various injection speeds.

Figure 13 illustrates the direct comparison of the lateral vibration displacement, force, and deformation at each speed. 

**Figure 14:**
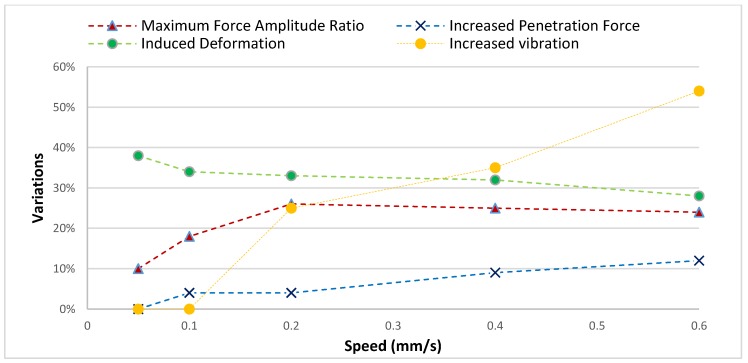
The impact of injection speed on lateral vibration displacement, penetration force, egg deformation, and force amplitude speeds.

Figure 14 illustrates the comparison between the force amplitude, penetration force, induced deformation and also increases in vibration. The maximum force amplitude ratio (MFAR) is defined as the ratio of the maximum force amplitude resultant during injection to the maximum penetration force in percentage. This demonstrates the force fluctuations during the injection. 0.05 mm/s injection speed is considered as the reference to calculate the increase in vibration displacement. As shown in Figure 14, the vibration displacement did not increase significantly at a lower speed, however, it increased significantly after 0.4 mm/s. The results demonstrate that force fluctuation increases while vibration displacements increases, however, it causes less deformation to the cell. The sudden force fluctuation was increased by 58% while lateral vibration displacement increased by up to 54% when the injection speed increased from 0.05 mm/s to 0.6 mm/s. The rate of increase for lateral vibration displacement and force fluctuations is consistent which demonstrates the reliability of the developed vision algorithm for vibration sensing. In the meantime, the egg deformation was reduced to approximately ~34%. Although the egg deformation was reduced, it has been ascertained that high lateral vibration displacement preventing egg injection should be conducted under stable conditions. The egg injection operation is challenging after 0.6 mm/s due to sudden increased lateral vibration displacement (up to 54%) which causes damage to the egg. Lateral vibration displacement is a decisive dynamic parameter in the egg-injection operation which limits the injection speed although increases in speed decrease the egg deformation in general trend. This demonstrates that injection operation requires speed optimization considering the induced vibration displacement as well as egg deformation. Overall, the correlation between the lateral vibration displacement, force fluctuation, egg deformation, and injection speed has demonstrated the critical role of visual vibration sensing in the ICSI operation.

## 4. Conclusions

In this paper, the vision-based three-dimensional vibrational motion detection technique was proposed for the application of biological cell injection. The advantage of this technique is that it uses only one microscope camera to obtain vibrational displacement information in three orthogonal axes. The developed vibration displacement measurement algorithm demonstrated consistency with the results obtained by ImageJ software. The vibration displacement occurred in the focal axis was also measured by the Brenner gradient algorithm. To show the significance of the developed vision algorithm, the effect of egg injection speed on lateral vibration displacement creation was investigated. It has been shown that the increase in injection speed resulted in an increase in the lateral vibration displacement at the tip of the injection pipette, which is up to 54% while the egg deformation has decreased to approximately 34%. It has further been demonstrated that the feasibility of egg injection is limited after the injection speed of 0.6 mm/s. The results show that lateral vibration displacement is a decisive parameter that needs to be measured in the egg injection task as it limits the injection speed. The proposed three-dimensional vibration sensor enhances the vibrational displacement measurement within the microinjections, specifically for ICSI. 

## Figures and Tables

**Table 1:** The average variation between Image J software and vision algorithm.

Level of Magnification	Axial Axis (µm) and Its Standard Deviation (SD)(µm)		Lateral Axis (µm) and Its Standard Deviation (SD)(µm)	
4×	0.808	0.841	1.05	0.881
10×	0.970	0.576	1.25	0.890
20×	0.790	0.302	0.46	0.264
40×	0.235	0.195	0.22	0.173
